# Analysis of lung cancer risk factors from medical records in Ethiopia using machine learning

**DOI:** 10.1371/journal.pdig.0000308

**Published:** 2023-07-19

**Authors:** Demeke Endalie, Wondmagegn Taye Abebe

**Affiliations:** 1 Faculty of Computing and Informatics, Jimma Institute of Technology, Jimma, Ethiopia; 2 Faculty of Civil and Environmental Engineering, Jimma Institute of Technology, Jimma, Ethiopia; National Yang Ming Chiao Tung University, TAIWAN

## Abstract

Cancer is a broad term that refers to a wide range of diseases that can affect any part of the human body. To minimize the number of cancer deaths and to prepare an appropriate health policy on cancer spread mitigation, scientifically supported knowledge of cancer causes is critical. As a result, in this study, we analyzed lung cancer risk factors that lead to a highly severe cancer case using a decision tree-based ranking algorithm. This feature relevance ranking algorithm computes the weight of each feature of the dataset by using split points to improve detection accuracy, and each risk factor is weighted based on the number of observations that occur for it on the decision tree. Coughing of blood, air pollution, and obesity are the most severe lung cancer risk factors out of nine, with a weight of 39%, 21%, and 14%, respectively. We also proposed a machine learning model that uses Extreme Gradient Boosting (XGBoost) to detect lung cancer severity levels in lung cancer patients. We used a dataset of 1000 lung cancer patients and 465 individuals free from lung cancer from Tikur Ambesa (Black Lion) Hospital in Addis Ababa, Ethiopia, to assess the performance of the proposed model. The proposed cancer severity level detection model achieved 98.9%, 99%, and 98.9% accuracy, precision, and recall, respectively, for the testing dataset. The findings can assist governments and non-governmental organizations in making lung cancer-related policy decisions.

## Author summary

Lung cancer has become one of the leading causes of mortality in Ethiopia. Lung cancer risk factors vary from place to place since it depends on the people’s socio-cultural activities. In this study, we examine lung cancer risk factors from the medical records of lung cancer patients in Addis Ababa, Ethiopia. The data contains the medical records of 872 women and 593 men. The key risk variables for lung cancer in the study area were identified using a decision tree. We discovered that coughing blood is one of the major risk factors for lung cancer, with a weight of 0.39. A feature importance of 0.39 indicates that the feature contributes 39% of the overall decision in the detection model. Furthermore, air pollution and obesity are the most important risk factors for lung cancer, with relevance weights of 0.21 and 0.14, respectively. This implies that these risk factors are causing or indicating most lung cancer cases in the study area. These three factors account for 74% of lung cancer analysis in the study area. Furthermore, we use the XGBoost classifier to detect lung cancer severity levels from risk factors, and the experiment yields a significant detection result.

## 1. Introduction

Cancer is a complex and diverse disease; its occurrence patterns vary according to variances in underlying cancer risk factors, such as environmental and lifestyle factors [1]. According to studies, cancer is on the rise in economically transitional countries due to rapid population growth, higher life expectancy, the adoption of unhealthy lifestyles, and changes in reproductive patterns [2]. The prevalence of cancer in Ethiopia is rapidly increasing, with an annual estimate of 77,352 new cancer cases in 2022 [3]. The cancer burden was estimated using the Addis Ababa population-based cancer registry. As a result, breast cancer (31.5%) and cervix cancer (14.1%) are the two most prevalent cancers among females, whereas colorectal cancers (10.6%) and non-Hodgkin lymphomas (10.2%) are the most common malignancies among males [4].

Lung cancer is the leading cause of cancer death worldwide, killing 1.8 million people each year. Only 20% of lung cancer cases are reported in low- and middle-income countries. An estimated 1.5% of all Ethiopian cancers involve the lungs [5]. Several risk factors for lung cancer have been identified by research [6]. Some of Ethiopia’s major lung cancer risk factors include smoking, alcohol use, passive smoking, air pollution, a family history of lung cancer (genetic risk), chest pain, and diet [7]. This study aimed to use a data mining algorithm to determine the lung cancer risk factor with the strongest relationship to its severity level and to build a model to predict the severity level from lung cancer risk factor records. Some of the works related to this study are listed below.

Authors in Ethiopia attempted to identify cancer symptoms and risk factors. For instance, the authors in [8] conducted a population-based face-to-face interview using a validated cancer awareness measure (CAM) method. A total of 600 adults (315 males and 285 females) were recruited utilizing a multistage sampling technique. One open-ended and ten closed-ended questions were used to test awareness of cancer. One open-ended and 12 closed-ended questions were used. The association between sociodemographic status and awareness of cancer signs, symptoms, and risk factors was investigated using logistic regression analysis. According to the closed questions responses, most respondents (80.7%) classified constant weariness as a cancer symptom and alcohol usage (82.5%) as a cancer risk.

The authors of [9] present a machine learning model for predicting factors for delayed BC diagnosis. In the study, four machine learning algorithms, including extreme gradient boosting (XGBoost), random forest (RF), neural networks (NNs), and logistic regression (LR), were used to examine the data of 630 women with confirmed BC. The most important factors for a delayed BC diagnosis were urban residency, breast disease history, other comorbidities, age at first childbirth, nulliparous ness, and being married. However, the significance of cancer risk factors is determined by their association with the severity degree [10].

The research of [11] considers 14 different possible risk factors for the two diseases ranging in importance from smoking and being overweight to drinking excessively hot drinks. Obesity has become a greater risk factor for Cancer and cardiovascular disease CVD in some areas, and research findings on some of these various factors correlated positively with their impact on disease burdens.

The work of [12] discusses using risk factors from various categories, such as epidemiology, radiology, and biomarkers, to target the population segment that will benefit the most from the newly introduced screening modality. Screening for lung cancer with low-dose computed tomography (LDCT) has been added to the arsenal of diagnostic tools available to people at high risk of developing the disease. While many pulmonary nodules are discovered, only a small percentage are early lung cancer. The vast majority of them are benign lesions of various types. Although the diagnostic work-up is time-consuming, the undeniable benefit stems from (I) lung cancer diagnosis at an earlier stage (stage shift); and (II) additional findings allowing the implementation of a preventive action that is not limited to thoracic oncology.

In [13], the authors proposed a model to predict the incidence of Colorectal Cancer (CRC) risk. The Clinical Cancer Research Nutrition database was used to drive and validate a model to predict CRC risk based on age and extended healthy lifestyle index components. The Soft-Voting classifier based on CatBoost, LightGBM, and Gradient-Boosting models provided an increased performance with an average accuracy of 0.6583 ± 0.054.

Data now drives more decisions than ever before. Data-based decisions are crucial for governmental and non-governmental organizations working in many fields. [14]. It reveals the hidden knowledge in their data. The health sector necessitates data-driven judgments [15]. As a result, the primary goal of this study is to use lung cancer patient’s medical records and information from healthy individuals who were tested for lung cancer as a control to make data-driven decisions about which lung cancer risk factor is particularly relevant in the study area. This study uses a decision tree algorithm to rank the risk factors from the hospital’s medical record. XGBoost machine learning is used to build a model that predicts the severity level of lung cancer. Finally, this study provides answers to the following questions:

1. Which risk factor is responsible for most lung cancer cases in the study area?
2. How can the level of lung cancer severity be detected using a machine learning model?

The entire work of this study is organized as follows: Section 2 covers the materials and methods used to achieve the study’s objective. Section 3 is about experiments and analyzing the results. Finally, Section 4 discusses the conclusion and future direction of this research.

## 2. Materials and Methods

**Fig 1:**
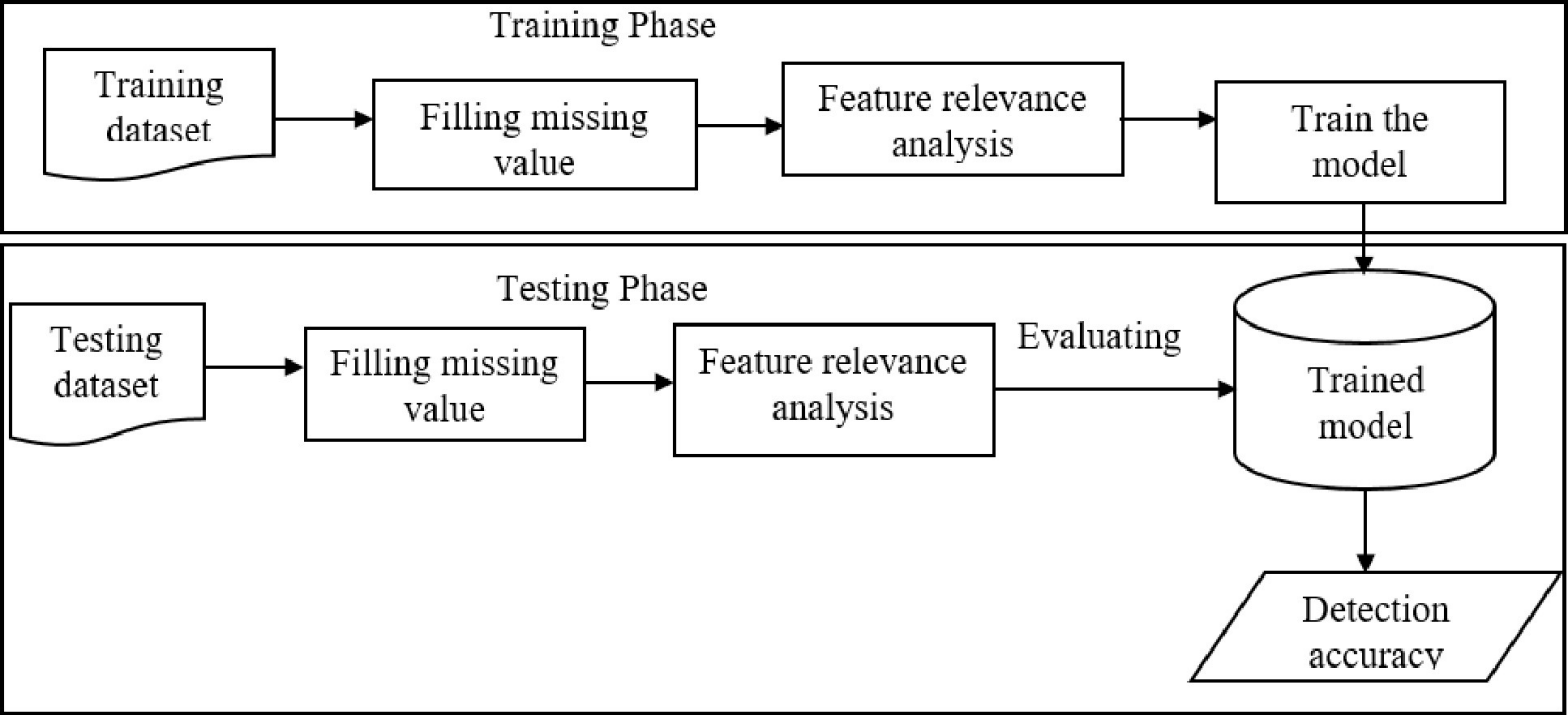
The architecture of the proposed cancer severity detection model.

The process of lung cancer risk factor analysis and a cancer severity detection model includes data collection, model evaluation, and model validation using various evaluation metrics. The high-level description of the proposed lung cancer severity detection model is shown in Fig 1. The architecture includes components such as a cancer patient’s demographic, medical history, and habits dataset; preprocessing components such as missing value filling, feature relevance calculation and selection; model training; and evaluation components.

The following is a description of each component of the proposed method.

### 2.1. Ethical Statements Approval

The study was approved by the Jimma University Institute of Health’s institutional review board, and we obtained permission from the Tikur Ambesa (Black Lion) Hospital administration and unit heads. Participants were told about the study’s aim, participation benefits, and their rights to stop at any time. We did not include the participant’s name or other personal information on the data collection sheets and reports.

### 2.2. Dataset Description

The data used for this study was compiled from patients’ medical records at Tikur Ambesa Hospital in Addis Abeba, Ethiopia. The medical record is a document that contains the following information: (1) the medical history, (2) the findings from the PE, (3) laboratory test reports, (4) the findings and conclusions from special examinations, (5) the findings and diagnoses of consultants, (6) the diagnoses of the responsible physician, (7) notes on treatment, including medication, surgical operations, radiation, and physical therapy, and (8) progress notes by physicians, nurses, and others [16].

This study includes medical records, including demographic information, habits, and medical histories of 1000 lung cancer patients with different severity levels and 465 healthy individuals who were checked for lung cancer. The quality and quantity of data found in their medical records are used to select participants for the study. There are 15 significant risk factors in the hospital setting; however, medical professionals classify 11 of the 15 as highly likely to be major risk factors for lung cancer. The severity of lung cancer is categorized into three levels, namely low, medium, and high, depending on the stage of the disease in the patient [17]. The risk factors were obtained from the medical records of the hospital. The data set contains the medical records of 872 women and 593 men. Participants in the study range in age from 14 to 73 years old. The total number of people in each of the four groups is shown in Table 1 below.

**Table 1:** The number of patients under each category of lung cancer severities.

Lung cancer severity category	Number of individuals in each category
Low	303
Moderate(middle)	332
High	365
Healthy	465

**Fig 2:**
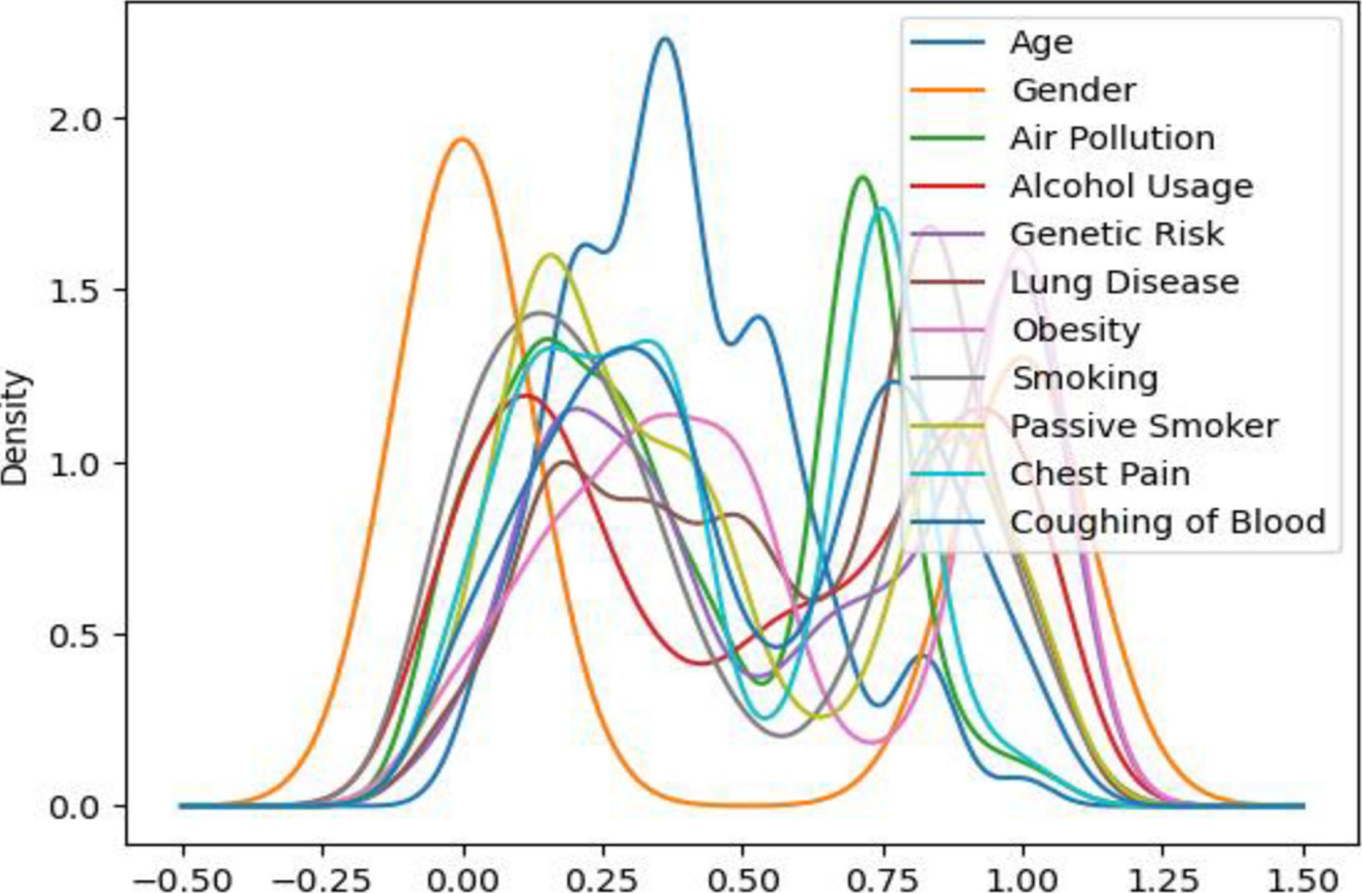
Distribution of values of each attribute in the dataset.

We used numeric codes to represent the values of individual risk factors obtained from medical records [218]. The lung cancer severity level is the dependent variable in our detection model and is determined by the degree of each risk factor. Fig 2 below depicts the normalized value distribution for each risk factor for lung cancer.

These characteristics are the risk factors that cause lung cancer with varying degrees of severity in Ethiopia. The study’s target or dependent variable is the lung cancer severity level (low, medium, high, and healthy). To process the data using any learning model, we convert the lung cancer severity levels to integers, converting low to 0, medium to 1, and high to 2. Three is the level we assign to healthy people. All of the learning models used in this study use those severity levels as a classification or prediction variable.

### 2.3. Filling Missing Values

The information collected in this research was cleansed and reviewed for outliers, mistakes, and missing. Missing data is a common issue in almost every real-world dataset. Missing values are defined as information about variables that are missing. The problem with missing values is that the analyses cannot be made correct based on the data, and the conclusions drawn from a dataset with missing values may be false [19]. The risk factors considered in this study are depicted in Table 2 below, along with the number of missed values for each risk factor (column).

**Table 2:** The number of missing values in each risk factor.

Risk factors	Missing rows under each risk factor
Age	0
Gender	0
Passive smoking	3
Smoking	6
Chest pain	12
Air pollution	4
Genetic risk	8
Alcohol usage	14
Obesity	6
Lung diseases	2
Coughing of blood	0
Total: 55 (3.75%)	

As shown in Table 2, we discovered a missing value in less than 4% of the records, so those rows with missing values can be removed [19]. Since eliminating reduces the quantity of the data, we used an imputation approach that takes a better evaluation of the data’s central tendency into consideration. We use the mode of each attribute value to fill in the missing values in the dataset [20]. We used mode to fill in the missing values in the dataset because all of the attributes in our dataset are categorical. Using mode values to impute missing data applies to numerical and categorical data [21]. We checked the mean and median of the values of each attribute before and after the change, and their difference was insignificant.

### 2.4. Feature Selection

Selecting a small subset of relevant features from the original features by removing irrelevant, redundant, or noisy features is known as feature selection [22]. Not all attributes or features are equally important for classification or detection problems. However, feature selection usually leads to better learning accuracy, lower computational cost, and better model interpretability. Before calculating the relationship between each attribute, we compute their skew values. Table 3 shows the outcome of the skewness value of each risk factor.

**Table 3:** Skew values for each lung cancer risk factor.

Risk factor	Skew value
Age	0.55
Gender	0.40
Air pollution	0.13
Alcohol Usage	-0.02
Genetic Risk	-0.13
Lung Disease	-0.22
Obesity	0.03
Smoking	0.38
Passive Smoker	0.40
Chest Pain	0.16
Coughing of Blood	0.12

The following is a rule of thumb for determining the distribution of data based on the skew value [23]:

✓ The distribution is highly skewed if the skewness is less than -1 or greater than 1.
✓ The distribution is moderately skewed if the skewness is between -1 and -0.5 or between 0.5 and 1.
✓ The distribution is approximately symmetric if the skewness is between -0.5 and 0.5.

According to the rule of thumb, only age has a moderate skew value; the others are classified as proximally symmetric. As a result, we use a correlation coefficient to determine the relationship between lung cancer risk factors.

Correlation coefficients measure the strength of the relationship between two variables [24]. The correlation coefficient can be expressed in terms of means and expectations:

$$Corr(fi,t)=E\frac{\left(fi-mean(fi))(t-mean(t)\right)}{\left(\sigma fi)(\sigma t\right)}$$ (1)

**Fig 3:**
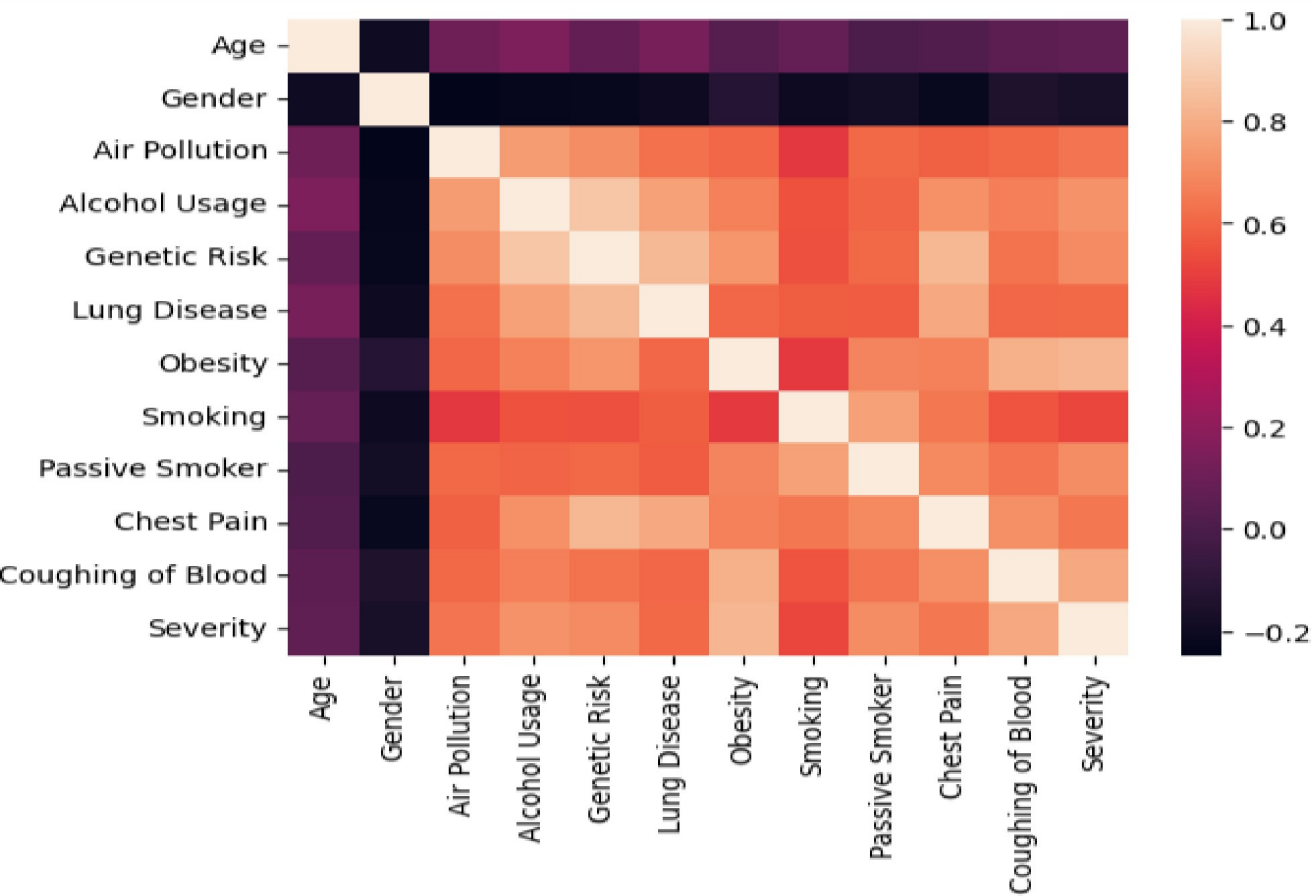
Correlation between each risk factor, including the dependent variable.

Where fi is feature i, E is the expectation, t is the target variable, σfi is the standard deviation of fi, and σt is the standard deviation of the target variable t. Fig 3 depicts the correlation between each risk factor investigated in this study. As a result, we evaluated the significance of the attributes listed in Table 2 above. We chose only those with a higher correlation coefficient with the target attribute or lung cancer severity level. The heat map in Fig 3 below substitutes numbers with colors of varied shades, as indicated by the scale on the right. Lighter cells have a higher correlation value. Looking at the relationship between the dependent variable (severity level) and the other independent variables, we can see that alcohol use, genetic risk, obesity, passive smoking, and blood coughing have the strongest positive correlation, while age and gender do not.

The correlation coefficient between -0.2 and 0.2 indicates that the two have an insignificant level of dependence in both positive and negative directions [25]. Depending on this, removing attributes with correlation coefficients between 0.2 and -0.2 does not affect a given learning model’s classification or predictive performance. As a result, when determining the severity level of lung cancer based on the above risk factors, we did not take a patient’s age or gender into account.

### 2.5. Train-test splitting

The train-test split module is used to estimate the performance of the proposed learning algorithm. It involves dividing the dataset into two subsets. The first subset is used to fit the model, and the second subset is supplied to the model after the prediction has been made and the result has been compared to the expected value. There is no globally accepted splitting ratio in machine learning [26]. However, Train: 80%, Test:20% is the most commonly used splitting percentage [27]. Therefore, we used an 80/20 train-test split ratio throughout the experiments. That means 80% of the data set is used to train the model, and 20% is used to test the trained model.

### 2.6. Learning Models

#### 2.6.1. Extreme Gradient Boosting

In this section, we will discuss the machine-learning methods that we used in our cancer severity detection system. The XGBoost classifier was chosen for our proposed cancer severity detection. XGBoost is a decision-tree-based ensemble machine learning algorithm that uses a gradient boosting algorithm before classifying a known dataset [28]. The two motivating factors that led us to choose XGBoost are model performance and execution speed. XGBoost is extremely fast when compared to other gradient-boosting implementations [29]. The mathematical explanation for XGBoost is provided below. XGBoost is made up of several Classification and Regression Trees (CART). According to CART, a basic decision tree can be established using the concept of entropy. CART’s target is the Gini coefficient [30]:

$$Obj:minGini(D,a)=\sum_{v=0}^{V}\frac{\left|D^{V}\right|}{\left|D\right|}Gini(D^{V}),$$

$$Gini(D)=1-\sum_{k=1}^{K}{\left(P_{k}\right)}^{2}$$ (2)

where a is one of the attributes we chose, V is the scale of a, v is one of the values of a, D is the dataset, P stands for the probability, and K is the label scale. Intuitively, the Gini coefficient reflects the likelihood that two samples in the dataset have different labels. Furthermore, this is the principle for building a single tree. The goal of XGBoost is to reduce the residual. The residual is the difference between the actual and predicted values.

#### 2.6.2. K-Nearest Neighbors (KNN)

KNN is a supervised machine-learning method commonly used for classification and regression applications. It is a non-parametric algorithm, which means it makes no assumptions about the data’s underlying distribution. The KNN algorithm begins by memorizing the full training dataset. When a new data point is submitted for classification or regression, the algorithm searches the training dataset for the K nearest neighbors based on some distance measure, where K is a user-defined value. The Euclidean distance is the most commonly used distance metric, but other distance metrics can also be utilized [31].

#### 2.6.3. Support Vector Machine

Support Vector Machine (SVM) is a supervised machine learning technique for classification and regression problems. A linear classifier seeks the hyperplane that best separates the input points into their respective classes. The SVM algorithm finds the hyperplane that maximizes the margin between the two nearest data points from distinct classes referred to as support vectors. The mathematical formulation of the SVM algorithm is as follows: Given an n-point training dataset D, the SVM method attempts to locate the hyperplane $w^{T}x+b=0$, which splits the data points into their respective classes. In this case, w is a weight vector, and b is a bias term. The hyperplane can be expressed as follows [32]:

$$w^{T}x_{i}+b\geq 1,if\;y_{i}=1\;or\;w^{T}x_{i}+b\leq -1,if\;y_{i}=-1$$ (3)

The SVM algorithm aims to maximize the margin while minimizing the classification error.

#### 2.6.4. Multilayer Perceptron

The Multilayer Perceptron (MLP) is an artificial neural network often used for supervised learning tasks such as classification and regression. It comprises numerous layers of interconnected neurons, where each neuron is a processing unit that receives input from the previous layer and outputs it to the next layer [33].

An MLP architecture typically consists of an input layer, one or more hidden layers, and an output layer. The input layer receives input data, which is subsequently processed by the hidden layers to form the network’s output. The output layer generates the network’s final output, a class label for classification tasks, or a numerical value for regression tasks. The MLP’s mathematical formulation is: Let X be the input data matrix of dimension n x p, where n is the number of data points, and p is the number of input features. Let Y be the output data matrix of dimension n x q, where q is the number of output classes or values. Let W be the size p x m weight matrix, where m is the number of neurons in the first hidden layer. Let V be the dimension m x q weight matrix, where q is the number of output classes or values. Let b1 be the size m x 1 bias vector for the first hidden layer. Let b2 be the output layer’s bias vector of size q x 1. The output of the first hidden layer is Z=f(XW+b1) [34].

Where f is the activation function, typically a nonlinear function such as the sigmoid or hyperbolic tangent function. The output of the output layer can be computed as Y_hat=softmax(ZV+b2), where softmax is a function used to convert the network output into a probability distribution over the output classes.

### 2.7. Evaluation Metrics

The models used to analyze the risk factors that cause cancer in Ethiopia can be measured in terms of detection accuracy, precision, recall, support, and confidence [35]. The following are the performance measures used in this study:

**Accuracy**: It may be defined as the number of correct predictions made as a ratio of all predictions made. We can easily calculate it using the confusion matrix and the following formula:

$$Accuracy=\frac{TP+TN}{TP+FP+FN+TN}$$ (4)

**Precision**: The proportion of correct positive predictions to total positive predictions. It is also known as positive predictive value.


$$Precision=\frac{TP}{TP+FP}$$ (5)

**Recall**: The proportion of correctly classified positive samples to total positive samples is represented by a recall. Similarly, specificity is defined as the proportion of correctly classified negative samples compared to total negative samples.


$$Recall=\frac{TP}{TP+FN}$$ (6)

Where True Positives (TP): It is the case when the data point’s actual and predicted class are 1. True Negatives (TN): It is the case when both the actual and predicted class of the data point are 0. False Positives (FP): It is the case when the actual class of data point is zero, and the predicted class of data point is 1. False Negatives (FN): It is the case when an actual class of data point is one, and the predicted class of data point is zero [36].

## 3. Experimental results and discussion

The experiment’s main goal was to detect the severity level of cancer disease from the risk factors that cause cancer. All of the experiments in this study were carried out on a computer with 16 GB of RAM, a Core i5, and the Windows 10 operating system. The source code for reading files, modeling, and presenting results was written in Python, and the hyperparameters of the machine learning algorithms used in this study were tuned using the gird search tuning strategy.

### 3.1. Risk factors analysis

Let us compute and identify the most influential risk factor for cancer in Ethiopia before developing a cancer severity detection model. The significance of features for cancer severity level is calculated using split points that improve the performance of the cancer severity detection measure and is weighted by the number of observations handled by the node [37]. The feature importance is built using the decision tree’s nodes and the features used to build the tree. Each node is assigned a split point on a given feature with an appropriate split criterion value. These values will be used to compute weight using the following formula [38]:

$$J(X_{j})=\sum_{n\in Q_{j}}split(n)$$ (7)

where Q_j_ denotes the set of nodes whose split point uses feature j and split(n) denote the value of the given split criterion for node n (depending on tree type). It should be noted that features that are not used in the tree are not included in the ranking and, as a result, have a weight of 0. The feature relevance is computed by a decision tree that uses the Gini splitting criterion. Gini impurity is one of the most popular and commonly used techniques that measure the impurity of the nodes and is calculated as [39]:

giniimpurity=1−Gini (8)

Considering that there are n classes, here is the sum of squares of success probabilities of each class and is given as:

$$Gini={(Pc_{1}}^{2}+{Pc_{2}}^{2}+{Pc_{3}}^{2}+{Pc_{n}}^{2})$$ (9)

**Fig 4:**
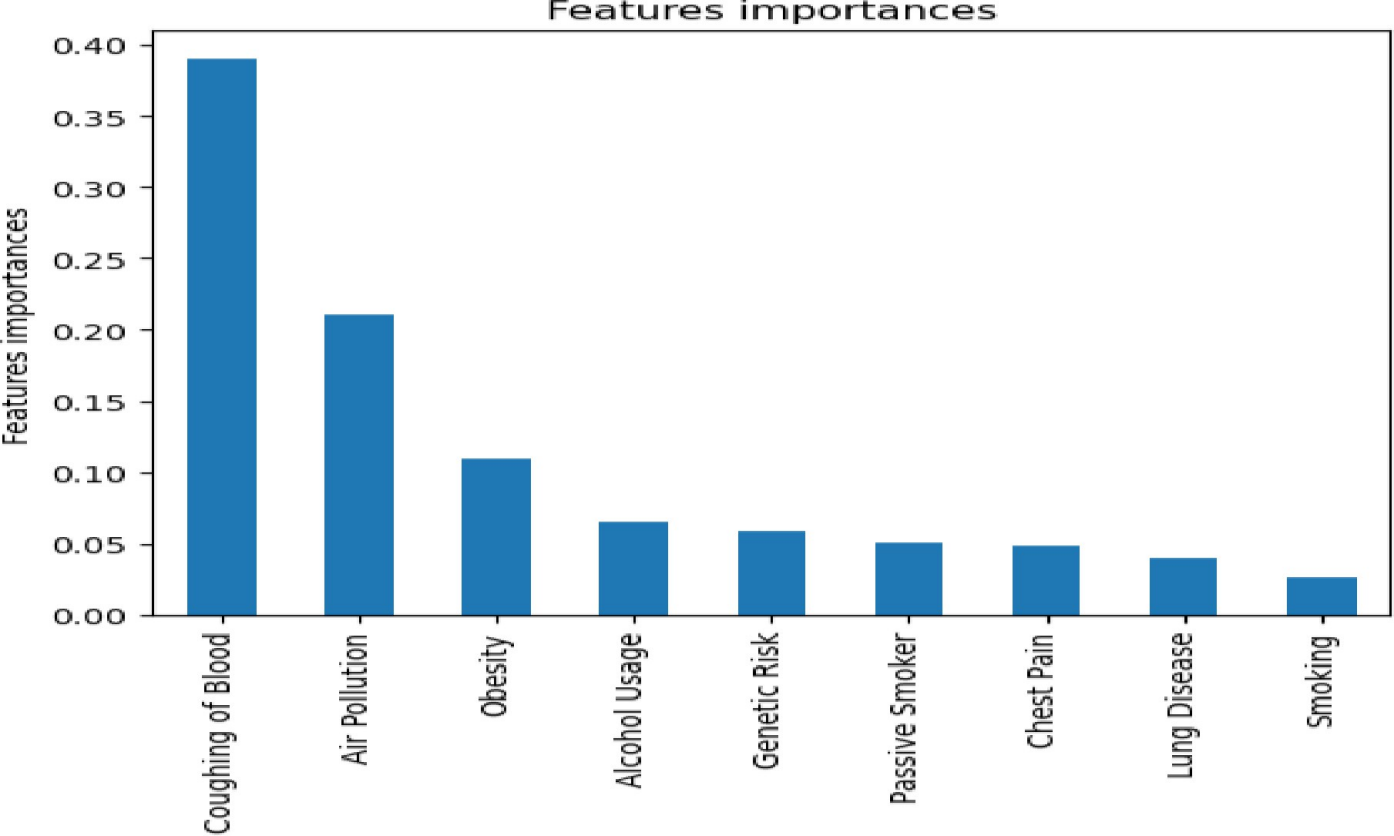
The importance of each lung cancer risk factor.

Then, the importance of each cancer risk factor in detecting cancer severity in the study area is depicted in Fig 4.

According to the results shown in Fig 4, the Coughing of blood is the leading risk factor of lung cancer because it is more relevant for detecting severity with a weight of 0.39. A feature importance of 0.39 indicates that the feature contributes 39% of the overall decision in the detection model. Furthermore, air pollution and obesity are the most important risk factors for lung cancer, with relevance weights of 0.21 and 0.14, respectively. This implies that these risk factors are causing or indicating most lung cancer cases in the study area. These three factors (blood coughing, air pollution, and obesity) account for 74% of lung cancer analysis decisions in the study area.

### 3.2. Cancer severity detection model

**Fig 5:**
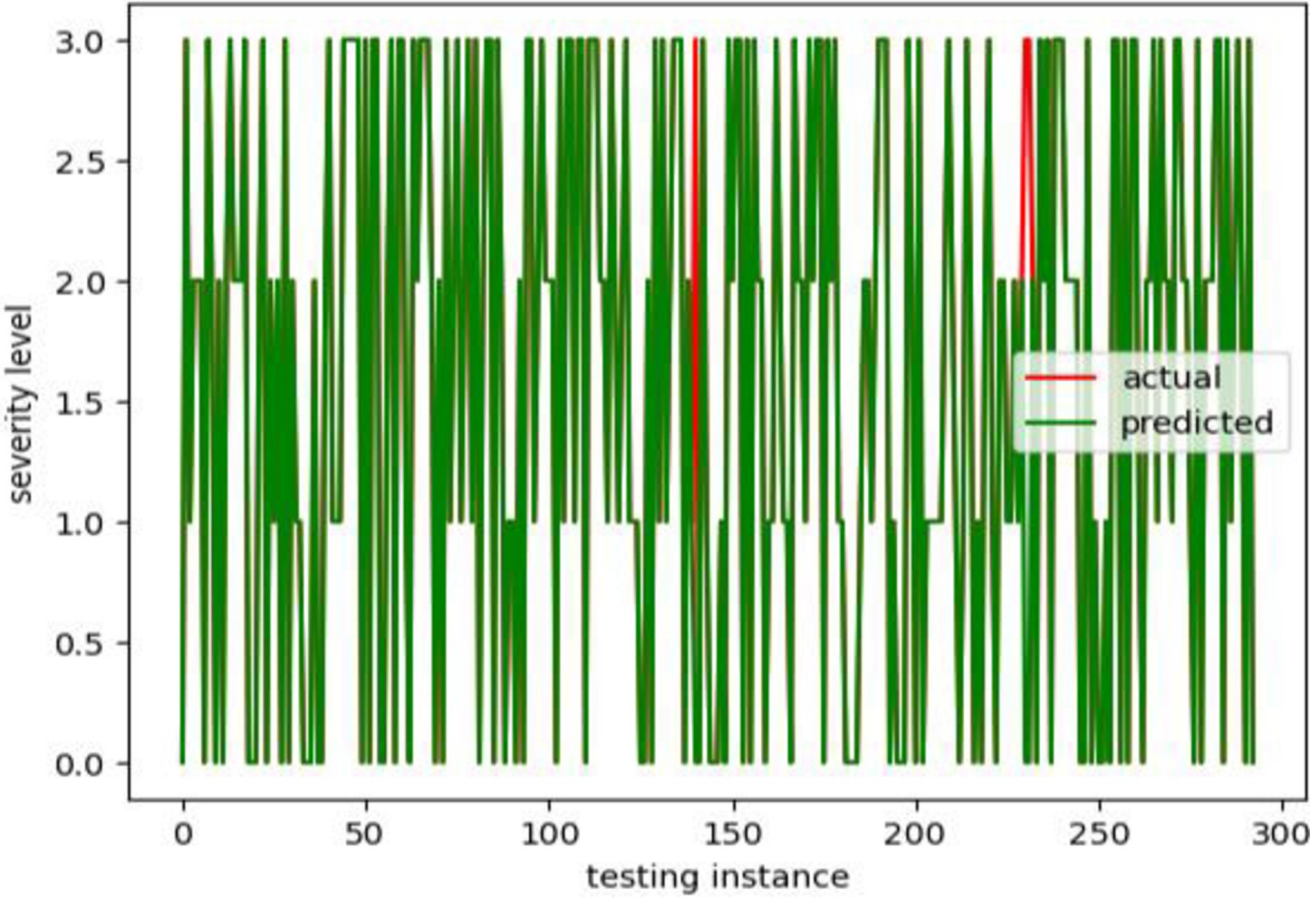
The actual and predicted cancer severity level for testing data.

The lung cancer severity detection model is created using an XGBoost classifier with an 80 percent data size for training and a 20 percent data size for testing the detection accuracy of the developed detection model. The model took nine attribute values as training and testing data and severity levels as training and testing labels. Fig 5 depicts the predicted and actual cancer severity levels and the overlap between the predicted and actual lung cancer severity levels. This means the proposed model correctly predicted the level for each testing instance.

Furthermore, the proposed cancer severity predictive model developed with the XGBoost algorithm is evaluated using various quality metrics such as accuracy, recall, precision, and confusion matrices. Table 4 shows the model’s accuracy, precision, and recall, and the results show that the proposed machine learning model detects the severity level of lung cancer by using nine demographics, habits, and medical histories of lung cancer patients and healthy individuals in Ethiopia.

**Table 4:** Evaluation of the proposed lung cancer severity detection model.

Model	Evaluation metrics		
XGBoost classifier	Accuracy	Precision	Recall
	98.9%	99%	98.9%

**Fig 6:**
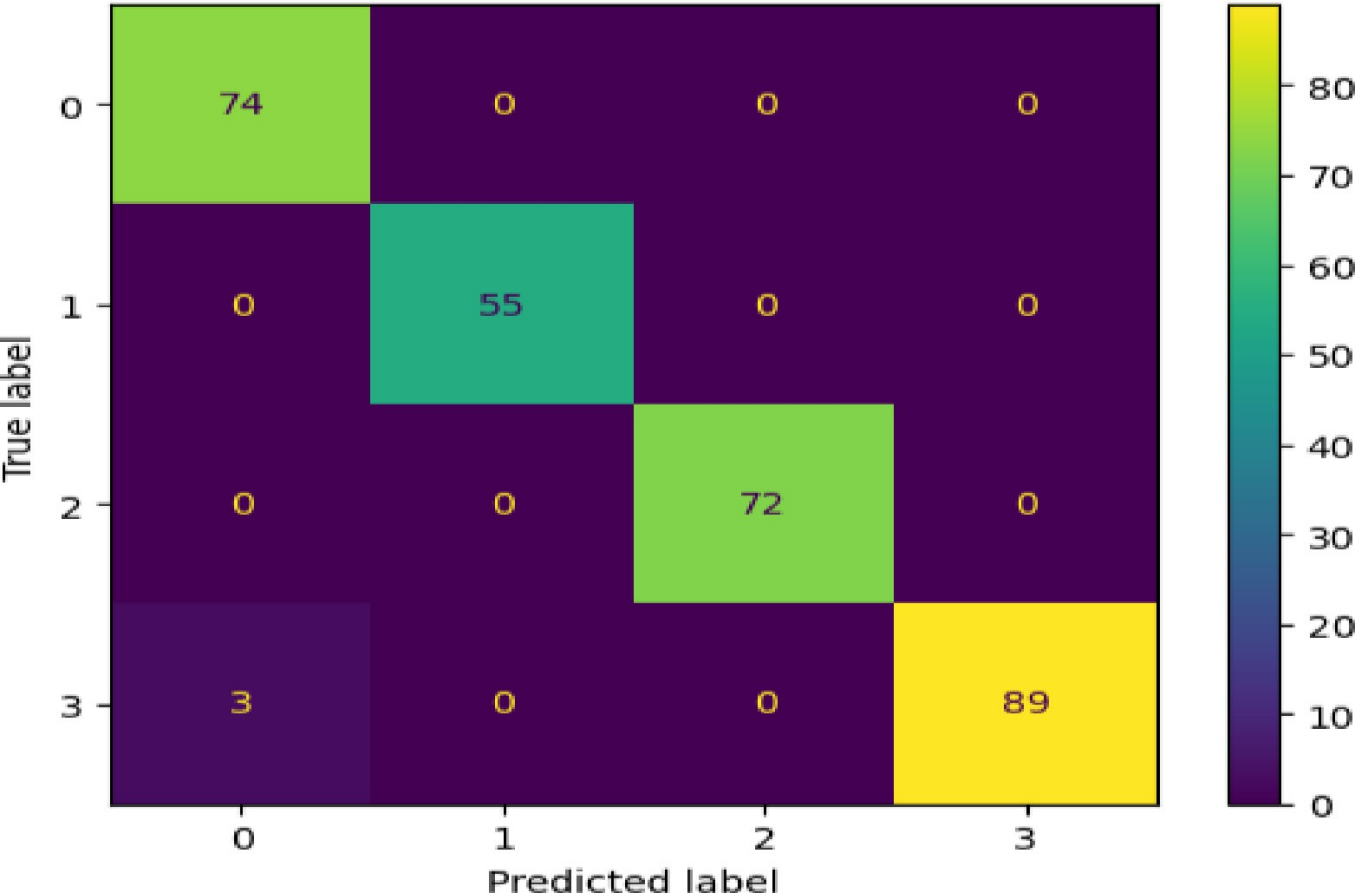
Confusion matrix produced by trained model.

Fig 6 depicts the confusion matrix for the proposed lung cancer severity detection model’s three severity levels, namely 0, 1, 2, and 3. We tested the model’s performance on 293 instances and correctly predicted 290. However, it predicted only three instances classified as "low-severity lung cancer "by doctors, and the model classified them as "healthy.”

Furthermore, we compare the proposed cancer severity level prediction model to machine learning models such as KNN, SVM, and MLP. The same dataset (our dataset) is used for comparison. When we ran learning models repeatedly, the outcomes varied [40]. However, by running the models ten times, we got the average values of each evaluation measure outcome. Table 5 compares each learning model in terms of the three quality measures. Regarding the three evaluation criteria, XGBoost outperforms KNN, SVM, and MLP.

**Table 5:** Comparison of XGBoost, MLP, KNN, and SVM with various evaluation metrics.

Learning Model	Performance		
	Accuracy	Precision	Recall
XGBoost	98.9%	99%	98.9%
MLP	93%	93.5%	93%
KNN	89%	90%	89%
SVM	90.4%	90.5%	90.4%

The number of instances incorrectly classified by each learning model is shown in Table 6 below. The table shows the number of incorrect classifications from the testing set out of 293 data instances (patients).

**Table 6:** Number of incorrectly classified instances by each learning model.

Learning model	Number of instances misclassified by models
XGBoost	3
MLP	20
KNN	32
SVM	28

According to the results in Table 6, the XGBoost classifier outperforms the other machine-learning algorithm by lowering the percentage of errors to 1%. As a result, the XGBoost classifier is chosen to create a lung cancer severity detection model for the research domain. The proposed model gives better results regarding the quality of the metrics used to evaluate its performance. Due to a data shortage, this study focused primarily on identifying the major lung cancer risk factor using a decision tree and developing a predictive model of lung cancer severity levels. However, in the future, we plan to expand it to include cancer classification and analysis of major risk factors for each type of cancer.

## 4. Conclusion

In this paper, we analyzed lung cancer risk factors and proposed a new lung cancer severity level predictive model. The data for this study came from Tikur Ambesa Hospital’s medical records repository, which included lung cancer patients and 465 healthy people who were tested for lung cancer as a control. We used a decision tree-based feature weighing strategy to determine which risk factor is dominant in the study area and the XGBoost machine learning algorithm to build a model to detect the severity level of lung cancer patients at the hospital. The results of the experiments suggest that dust allergies, obesity, fatigue, alcohol use, and passive smoking are the most prevalent risk factors in the study area. In addition, the proposed cancer severity level detection model produces an acceptable result with higher detection accuracy. Therefore, the findings of this study deserve to be used in different applications that use the severity level of cancer or in making health policies related to cancer. The study will be expanded with more data in the future, and it will be one component of a system that notifies the severity level of lung cancer based on risk factors.
